# Enhancement of carotenoid production by disrupting the C22-sterol desaturase gene (*CYP61*) in *Xanthophyllomyces dendrorhous*

**DOI:** 10.1186/1471-2180-12-235

**Published:** 2012-10-18

**Authors:** Iris Loto, María Soledad Gutiérrez, Salvador Barahona, Dionisia Sepúlveda, Pilar Martínez-Moya, Marcelo Baeza, Víctor Cifuentes, Jennifer Alcaíno

**Affiliations:** 1 Laboratorio de Genética. Departamento de Ciencias Ecológicas y Centro de Biotecnología, Facultad de Ciencias, Universidad de Chile, Las Palmeras 3425, Santiago Casilla 653, Chile

**Keywords:** *Xanthophyllomyces dendrorhous*, Astaxanthin, Ergosterol, Sterol C22-sterol desaturase, Cytochrome P450

## Abstract

**Background:**

*Xanthophyllomyces dendrorhous* is a basidiomycetous yeast that synthesizes astaxanthin, which is a carotenoid with a great biotechnological impact. The ergosterol and carotenoid synthesis pathways are derived from the mevalonate pathway, and in both pathways, cytochrome P450 enzymes are involved.

**Results:**

In this study, we isolated and described the *X. dendrorhous CYP61* gene, which encodes a cytochrome P450 involved in ergosterol biosynthesis. This gene is composed of nine exons and encodes a 526 amino acid polypeptide that shares significant percentages of identity and similitude with the C22-sterol desaturase, CYP61, from other fungi. Mutants derived from different parental strains were obtained by disrupting the *CYP61* gene with an antibiotic selection marker. These mutants were not able to produce ergosterol and accumulated ergosta-5,8,22-trien-3-ol and ergosta-5,8-dien-3-ol. Interestingly, all of the mutants had a more intense red color phenotype than their respective parental strains. The carotenoid composition was qualitatively and quantitatively analyzed by RP-HPLC, revealing that the carotenoid content was higher in the mutant strains without major changes in their composition. The expression of the *HMGR* gene, which encodes an enzyme involved in the mevalonate pathway (3-hydroxy-3-methylglutaryl-CoA reductase), was analyzed by RT-qPCR showing that its transcript levels are higher in the *CYP61* mutants.

**Conclusions:**

These results suggest that in *X. dendrorhous*, ergosterol regulates *HMGR* gene expression by a negative feedback mechanism and in this way; it contributes in the regulation of the carotenoid biosynthesis.

## Background

*Xanthophyllomyces dendrorhous* is a basidiomycetous carotenogenic yeast and is one of the few known natural sources of xanthophyll astaxanthin (3,3’-dihydroxy-β,β-carotene-4-4’-dione) [1-3]. Carotenogenesis may have evolved as a cellular defense mechanism against oxidative damage from reactive oxygen species (ROS) produced by biochemical and photochemical systems [4-6]. Among carotenoids, astaxanthin stands out for its potent antioxidant properties and other beneficial effects on human health [7]. Moreover, this pigment has been widely used in aquiculture to color the flesh of cultured salmonids. Because the characteristic pigmentation is highly desired by consumers, astaxanthin availability has an impact on production costs [8]. Due to its prevalent use in the food, aquiculture, pharmaceutical and cosmetic industries and the increasing demand for natural products, astaxanthin and its sources have great commercial potential [2,8].

**Figure 1:**
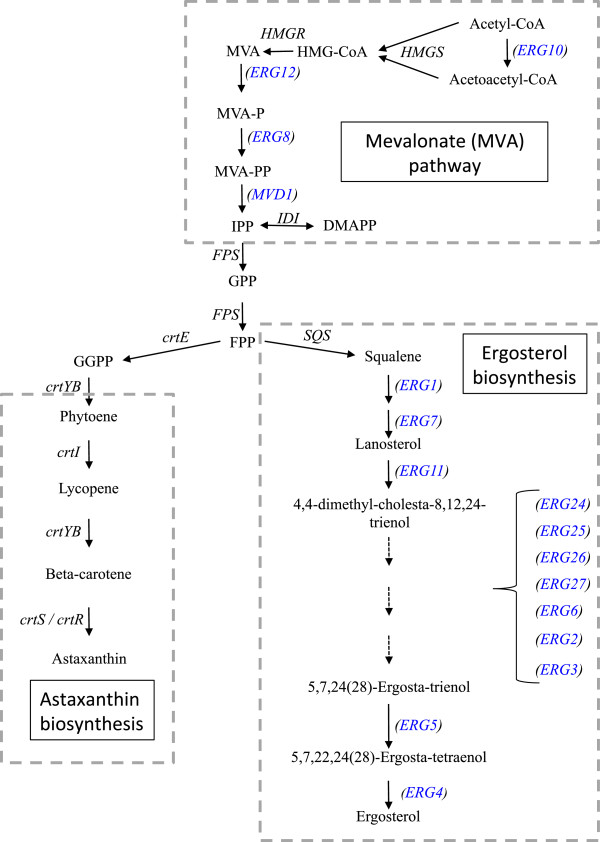
**Mevalonate pathway**, **astaxanthin and ergosterol biosynthesis.** The arrows represent the catalytic step with the respective enzyme-encoding gene described in *X. dendrorhous* (gene names without brakets and written in black) and *S. cerevisiae* (genes between brackets and written in blue). The represented *X. dendrorhous* genes with their Genbank accession number in square brackets are: *HMGR* [AJ884949], *IDI* [DQ235686], *crtE* [DQ012943], *crtYB* [DQ016503], *crtI* [Y15007], *crtS* [EU713462] and *crtR* [EU884133]. The *X. dendrorhous HMGS*, *FPS* and *SQS* gene sequences are submitted in patents [DI059433.1, DI032788.1 and EA489199, respectively]. The following *S. cerevisiae* genes are represented: *ERG10* [NM_001183842], *ERG13* [NM_001182489], *ERG12* [AN: NM_001182715], *ERG8* [NM_001182727], *MVD1* [NM_001183220], *ERG20* [NM_001181600], *ERG1* [M64994], *ERG7* [U23488.1], *ERG11* [NM_001179137], *ERG24* [NM_001183118], *ERG25* [NM_001181189], *ERG26* [NM_001180866], *ERG27* [NM_001181987], *ERG6* [NM_001182363], *ERG2* [NM_001182709], *ERG3* [NM_001181943], *ERG5* [NM_001182511], and *ERG4* [NM_001180877]. Abbreviations: 3-hydroxy-3-methylglutaryl-CoA (HMG-CoA), mevalonate (MVA), mevalonate-5-phosphate (MVA-P), mevalonate-5-pyrophosphate (MVA-PP), isopentenyl-pyrophosphate (IPP), dimethylallyl-pyrophosphate (DMAPP), geranyl-pyrophosphate (GPP), farnesyl-pyrophosphate (FPP), geranylgeranyl-pyrophosphate (GGPP).

Carotenoids are tetraterpenoid compounds that are biosynthesized in the isoprenoid (also known as terpenoid) pathway (Figure 1); the basic units are isopentenyl-pyrophosphate (IPP) and its isomer dimethylallyl-pyrophosphate (DMAPP) [9]. Although an alternate pathway has been described (the deoxyxylulose phosphate, methylerithritol phosphate, or nonmevalonate pathway), IPP is synthesized from acetyl-CoA via the mevalonate (MVA) pathway in most eukaryotes [10]. Five genes control this pathway, and among them, the expression of the gene that encodes hydroxymethylglutaryl-CoA (HMG-CoA) reductase, *HMGR,* is strongly regulated at different levels (transcription, post-translational and proteolysis) [11]. In the isoprenoid synthesis pathway (Figure 1), DMAPP and IPP are condensed by prenyl transferases to form geranyl-pyrophosphate (GPP), and the addition of a second molecule of IPP gives rise to farnesyl pyrophosphate (FPP) [9]. Squalene, the precursor of sterols, is formed by the condensation of two molecules of FPP by squalene synthase [12]. For the biosynthesis of carotenoids, a third IPP unit is added to FPP, generating geranylgeranyl-pyrophosphate (GGPP). The condensation of two molecules of GGPP forms the first carotenoid in this biosynthetic pathway, phytoene [13]. During *X. dendrorhous* carotenogenesis, lycopene is formed by four successive desaturations of phytoene; cyclization of the ends of lycopene produces beta-carotene [14]. Unlike other astaxanthin-producing organisms, *X. dendrorhous* has a single astaxanthin synthase (encoded by the *crtS* gene) that catalyzes the ketolation and hydroxylation of beta-carotene to produce astaxanthin [15,16]. This enzyme is related to a 3A sub-family member of the cytochrome P450 protein family [15,16]. Cytochrome P450 proteins (P450s) are heme-containing monooxygenases that are present in organisms from all domains of life [17]; P450s have significant roles in the oxidative metabolism of many exogenous and endogenous substrates [18]. In their active state, these enzymes are reduced by electrons that are supplied by NAD(P)H through a P450 redox partner [19], which in eukaryotes is a cytochrome P450 reductase [20]. In *X. dendrorhous*, the *crt*R gene encodes the yeast cytochrome P450 reductase that is essential for the synthesis of astaxanthin [21]. However, the *X. dendrorhous crtR* gene is different from the *crtR* gene originally described in cyanobacterium *Synechocystis* sp., which encodes a beta-carotene hydroxylase [22].

Sterols and carotenoids are derived from IPP. Sterols are essential structural and regulatory components of eukaryotic cell membranes, modulating their thickness, fluidity and permeability [23]. Ergosterol is the principal sterol in yeasts, and two cytochrome P450s are involved in its biosynthesis: CYP51 (lanosterol 14-demethylase) and CYP61 (C-22 sterol desaturase), which in *Saccharomyces cerevisiae* are encoded by the *ERG11* and *ERG5* genes, respectively [24] (Figure 1). An *erg*5^-^*S. cerevisiae* mutant strain is viable but unable to synthesize ergosterol [25]. Interestingly, one of the major bottlenecks in ergosterol biosynthesis is the reaction catalyzed by HMG-CoA reductase [26]. As MVA is a common precursor in ergosterol and carotenoid biosynthesis, its synthesis should also be an important bottleneck in the biosynthesis of astaxanthin in *X. dendrorhous*. Based on these observations, this study aimed to identify and characterize the *X. dendrorhous* C-22 sterol desaturase encoding gene, *CYP61*, and to evaluate the effect of its disruption on yeast ergosterol production and carotenogenesis.

## Results

### Cloning and sequence analysis of the *CYP61* gene from *X. dendrorhous*

Our *X. dendrorhous* genomic database was analyzed with the BLAST tool of the CLC Genomics Workbench 5 software using as query several *CYP61* gene sequences available in the GenBank database. In this way, we were able to identify a putative *CYP61* gene (hereafter *CYP61* gene) from *X. dendrorhous*, which allowed us to design specific primers to amplify and clone this gene.

**Figure 2:**
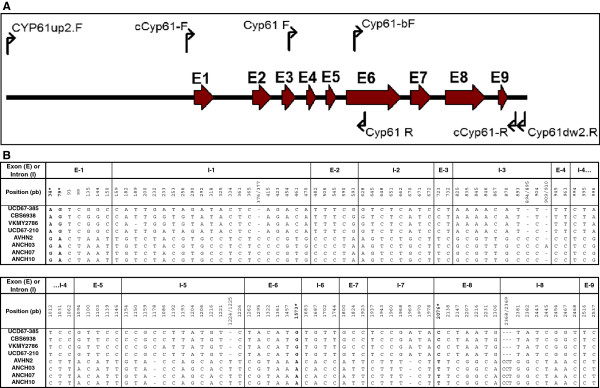
**Structure and sequence comparison of the *CYP61 *gene from *X****.****dendrorhous.*** (**A**) PCR-amplified DNA region that includes the *CYP61* gene from *X. dendrorhous*. The nine exons of the *CYP61* gene are shown in thick red arrows (E1 to E9) and the recognition site of primers used in this work are represented by thin arrows. (**B**) Sequence comparison of *CYP61* genes from different *X. dendrorhous* strains: UCD 67–385 [GenBank: JX183236], CBS 6938 [GenBank: JX183240], VKM Y-2786 [GenBank: JX183238], UCD 67–210 [GenBank: JX183237], AVHN2 [GenBank: JX183239], ANCH03 [GenBank: JX183241], ANCH07 [GenBank: JX183242] and ANCH10 [GenBank: JX183243]. The base changes are shown indicating their position in bp, with the adenine of the translation start codon as bp 1. The respective exon (E-1 to E-9) or intron (I-1 to I-8) where the base changes occur is also indicated.

A fragment of approximately 4,200 bp [GenBank: JX183236] was PCR-amplified using genomic DNA from strain UCD 67–385 as a template and the primer set CYP61up2.F + CYP61dw2.R (Table 1). This fragment was inserted at the *Eco*RV site of the pBluescript SK- plasmid, generating pBS-gCyp61. In parallel, the *X. dendrorhous CYP61* cDNA was screened in a cDNA library by PCR using plasmid DNA from different clone mixtures as templates and the primer pair CYP61.F + CYP61.R (Table 1). The recombinant plasmid pBS-cCyp61, which contained the *CYP61* gene cDNA with an ORF of 1,581 bp [GenBank: JX183235], was isolated. The sequence analysis of the genomic and cDNA versions of the *CYP61* gene allowed us to determine that this gene consists of nine exons of 156, 152, 114, 75, 81, 441, 169, 320 and 73 bp, and eight introns of 317, 82, 90, 83, 84, 79, 116 and 111 bp (Figure 2A). The *CYP61* gene encodes a putative 526 amino acid CYP61 protein with a predicted molecular weight of 59.6 kDa and p*I* of 6.48. The CYP61 deduced protein from *X. dendrorhous* shares 43% identity and 65% similarity at 95% sequence coverage with the *Saccharomyces cerevisiae* C22-sterol desaturase (CYP61, Swiss-Prot: P54781.1). This protein belongs to the cytochrome P450 protein family and is involved in the second last step of the ergosterol biosynthesis, the conversion of 5,7,24(28)-ergostatrienol into 5,7,22,24(28)-ergostatetraenol [25].

**Table 1:** Primers designed and used in this work

**Nº**	**Primer**	**Sequence 5**’ **to 3**’	**Target**
1	H-out.F	CTCGATGAGCTGATGCTTTG	Hygromycin B resistance cassette
2	H-out.R	TCCATCACAGTTTGCCAGTG	Hygromycin B resistance cassette
3	Zeo.F	TGAACAGGGTCACGTCGT	Zeocin resistance cassette
4	Zeo.R	CGCTGATGAACAGGGTCAC	Zeocin resistance cassette
5	CYP61up2.F	CTGGAGCCGAATTCATTGAT	*CYP61* gene
6	CYP61dw2.R	AGGAGGCAGAGTGGTTGAGA	*CYP61* gene
7	CYP61b.F	GTCGGAGGAAGAGCAGTTTG	*CYP61* gene
8	CYP61.F	CTGAGCCCTGTCTTGTTGCC	*CYP61* gene
9	CYP61.R	ATTGTACACCTTTGTTCCAGGC	*CYP61* gene
RT-qPCR (The pairs of primers used had efficiency greater than 95%, as determined by standard curves with a correlation coefficient of R2 ≥ 0.996):			
10	mactF-RT	CCGCCCTCGTGATTGATAAC	*ACT* gene
11	mactR-RT	TCACCAACGTAGGAGTCCTT	*ACT* gene
12	hmgR.F-RT	GGCCGATCGCTATACATCCGTTT	*HMGR* gene
13	hmgR.R-RT	ATCCAGTTGATGGCAGAAGGCT	*HMGR* gene

F and R in the primer name indicate the primer orientation.

The *CYP61* gene from several *X. dendrorhous* strains (VKM Y-2786, CBS 6938, UCD 67–210, ANCH03, ANCH07, ANCH10 and AVHN2 (Table 2) was PCR-amplified using *Pfu* DNA pol, and each amplicon was sequenced [GenBank: JX183238, JX183240, JX183237, JX183241, JX183242, JX183243 and JX183239, respectively]. We found several base changes, but most of them were located in the intronic regions. Only four base changes produced amino acid replacements; the adenine, guanine, guanine and cytosine at positions 34, 79, 1,573 and 2,075 were converted to guanine, adenine, adenine and thymine (numeration according to the *CYP61* gene translation start in strain UCD 67–385), resulting in T12A, A27T, R306K and P409S variations at the deduced amino acid sequence, respectively (Figure 2B).

**Table 2:** Strains and Plasmids used and built in this work

	**Genotype or relevant features**	**Source or reference**
Strains:		
*E. coli*:		
DH-5α	F- φ80d lacZΔM15Δ (lacZY-argF) U169 deoR recA1 endA1 hsdR17(rk- mk+) phoA supE44l- thi-1 gyrA96 relA1	[52]
*X. dendrorhous*:		
UCD 67-385	ATCC 24230, wild type.	ATCC
	Diploid strain [30]	
385-*cyp61*^(+/−)^	(385-*CYP61*/*cyp61*^*hph*^). Heterozygote transformant derived from UCD 67–385 containing an allele of the *CYP61* locus interrupted with a hygromycin B resistance cassette.	This work
385-*cyp61*^(−/−)^	(385-*cyp61*^*hph*^/*cyp61*^*zeo*^). Homozygote transformant derived by transformation of 385-*cyp61*^+/−^ with both *CYP61* alleles interrupted, one with a hygromycin B resistance cassette and the other with a zeocin resistance cassette.	This work
CBS 6938	ATCC 96594, wild type.	ATCC
CBS-*cyp61*^(−)^	(CBS-*cyp61*^*hph*^). Hemizygote transformant derived from CBS 6938. The single *CYP61* locus was interrupted with a hygromycin B resistance cassette.	This work
AVHN2^*^	Chilean native isolate, wild type.	Our Lab collection
Av2-*cyp61*^(−)^	(Av2-*cyp61*^*zeo*^). Hemizygote transformant derived from AVHN2. The single *CYP61* locus was interrupted with a zeocin resistance cassette.	This work
UCD 67-210	ATCC 24202, wild type (*Phaffia rhodozyma*)	ATCC
VKM Y-2786	Wild-type strain.	VKM
ANCH03^*^	Chilean Antarctic native isolate, wild type.	Our Lab collection
ANCH07^*^	Chilean Antarctic native isolate, wild type.	Our Lab collection
ANCH10^*^	Chilean Antarctic native isolate, wild type.	Our Lab collection
Plasmids:		
pBluescript SK- (pBS)	ColE1 ori; AmpR; cloning vector with blue-white selection	Stratagene
pMN-*hph*	pBS containing at the *Eco*RV site a cassette of 1.8 kb bearing the *E. coli*-Hygromycin B resistance (*hph*) gene under EF-1 α promoter and GPD transcription terminator of *X. dendrorhous*.	[31]
pIR-*zeo*	pBS containing at the *Eco*RV site a cassette of 1.2 kb bearing the *Streptoalloteichus hindustanus* Zeocin resistance *Sh ble* gene under EF-1 α promoter and GPD transcription terminator of *X. dendrorhous*.	This work
pBS-gCyp61	pBS containing at the *Eco*RV site a 4,224 bp DNA fragment containing the *X. dendrorhous CYP61* gene amplified by PCR with primers CYP61up2.F and CYP61dw2.R.	This work
pBS-cyp61/Hyg	pBS-gCyp61 bearing the Hygromycin B resistance cassette at the *Eco*RV site that interrupts the *CYP61* gene.	This work
pBS-cyp61/Zeo	pBS-gCyp61 bearing the Zeocin resistance cassette at the *Eco*RV site that interrupts the *CYP61* gene.	This work
pBS-cCyp61	pBS bearing the cDNA of the *CYP61* gene. The cDNA measures 1,752 bp with an ORF of 1,581 bp.	This work

^*^: *X. dendrorhous* Chilean native isolates confirmed by ITS, D1/D2 and IGS regions sequences. The following abbreviations are used for microorganism culture collections: CBS, Centraalbureau voor Schimmelcultures, Utrecht, Netherlands; ATCC, American Type Culture Collection, Manassas, USA; UCD, Phaff Yeast Culture Collection, Department of Food Science and Technology, University of California at Davis, Davis, USA; VKM, The All-Russian Collection of Microorganisms, Moscow, Russia.

**Figure 3:**
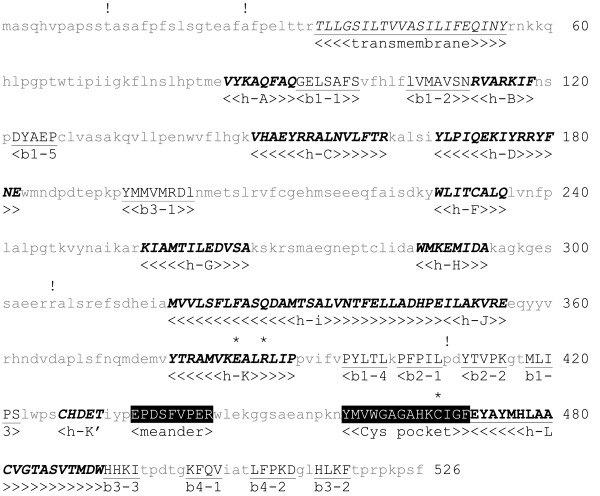
**Deduced *X****.****dendrorhous *CYP61.** Secondary structural elements were predicted with the CYP450 Engineering database, and the potential transmembrane region was predicted with TMpred. Structural elements are in capital letters with the name of the corresponding feature underneath them. Underlined and in italics: possible transmembrane helix. In bold and italics: alpha helices. Underlined: Beta-sheets. In white letters and highlighted in black: meander loop and Cys pocket. The asterisks (*) indicate the three totally conserved amino acids among cytochromes P450, and the exclamation points (!) show the amino acid variation found in the deduced CYP61 from different *X. dendrorhous* strains.

Even though the amino acid sequences are extremely diverse among the cytochrome P450 protein family, their structural fold is highly conserved [27]. Several cytochrome P450 secondary structural elements in the deduced CYP61 protein from *X. dendrorhous* were predicted with the CYP450 Engineering database [28] (Figure 3). This included alpha helices A, B, C, D, F, G, H, I, J, K, K’ and L, beta-sheets 1–1, 1-2, 1–5, 3–1, 1–4, 2–1, 2–2, 1–3, 3–3, 4–1, 4–2 and 3–2, the meander loop, which may be involved in the stabilization of the tertiary structure and heme binding, and the Cys pocket that contains the conserved cysteine involved in heme binding. There are three totally conserved amino acids in the cytochrome P450 protein family, the glutamic acid and arginine of the E-X-X-R motif at the K-helix, which are involved in stabilizing the core and heme binding, and the heme binding cysteine [28], and these residues are present in the predicted CYP61 protein. Additionally, we were able to predict the putative hydrophobic transmembrane segment at the CYP61 amino terminus, which could anchor the protein to the endoplasmic reticulum [29].

### The *CYP61* gene mutation

**Figure 4:**
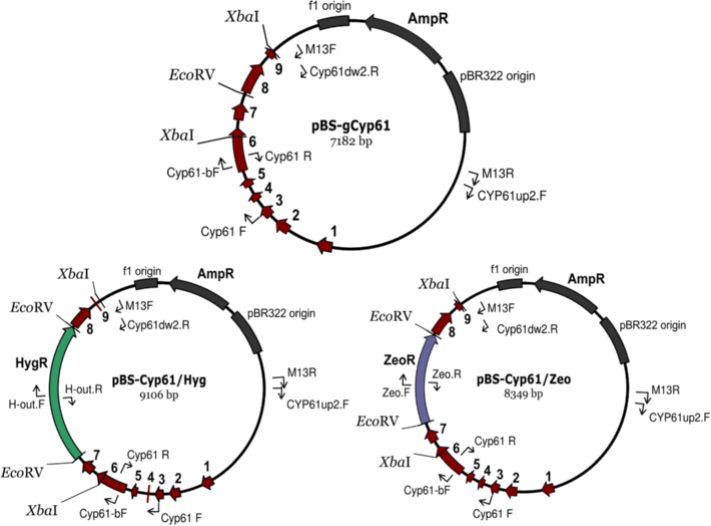
**Plasmids constructed in this work.** In each plasmid illustration, relevant features for this work, such as endonuclease recognition sites and primer binding sites (thin arrows), are shown. Some elements of the original plasmid (pBluescript SK-) were kept and shown in gray. Plasmid pBS-gCyp61 harbors the genomic version of the *CYP61* gene from *X. dendrorhous* that was PCR-amplified with primers CYP61up2.F and Cyp61dw2.R. Red thick arrows along with a number represent the nine exons of the *CYP61* gene. Plasmids pBS-Cyp61/Hyg and pBS-Cyp61/Zeo were built by inserting the hygromycin B (HygR, in green) and zeocin (ZeoR, in violet) resistance expression cassettes, respectively, at the *Eco*RV site of plasmid pBS-gCyp61. To linearize the plasmids for transformation purposes, pBS-Cyp61/Hyg and pBS-Cyp61/Zeo were digested with *Xba*I.

**Figure 5:**
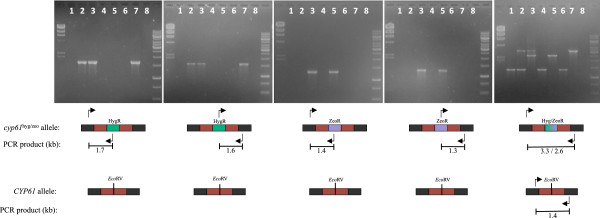
**PCR**-**based analysis of *cyp61***^*-*^**mutants.** Each gel shows PCR reactions performed with different sets of primers and genomic DNA from strains UCD 67–385 (lane 1), 385-*CYP61*/*cyp61*^*hph*^ (lane 2), 385-*cyp61*^*hph*^/*cyp61*^*zeo*^ (lane 3), CBS 6938 (lane 4), CBS*-cyp61*^*hph*^(lane 5), AVHN2 (lane 6), Av2*-cyp61*^*zeo*^ (lane 7), and a negative control without DNA (lane 8). The diagram below each gel represents the amplification target (*cyp61*^*-*^ mutant or wild-type allele) and the size of the expected amplicon. The colors represent the resitance cassettes HygR in green and ZeoR in violet, the *CYP61* gene in red and the *CYP61* flanking DNA in dark grey. The *Eco*RV recognition site, where the respective antibiotic resistance marker was inserted to disrupt the *CYP61* gene, is also shown. Molecular weight standard were: lambda DNA/*Hind* III (23.1, 9.4, 6.6, 4.4, 2.3, 2.0 and 0.6 kbp) at the left, and 1 kb DNA ladder (10.0, 8.0, 6.0, 5.0, 4.0, 3.5, 3.0, 2.5, 2.0, 1.5, 1.0, 0.75 and 0.5 kbp) at the right, of each gel.

To study the function of the *CYP61* gene in *X. dendrorhous*, mutant *cyp61*^*-*^ strains were generated. The wild-type strains UCD 67–385 and CBS 6938 were transformed with plasmid pBS-cyp61/Hyg, and strain AVHN2 was transformed with plasmid pBS-cyp61/Zeo. All transformations were performed with linearized plasmids as indicated in Figure 4. Through a double homologous recombination event, the donor DNA fragment containing the *CYP61* gene interrupted by one of the two resistance markers replaced the *CYP61* gene in the yeast chromosome. In this way, we obtained the transformant strains 385-*cyp61*^*hph*^*,* CBS*-cyp61*^*hph*^ and Av2*-cyp61*^*zeo*^ (Table 2). The genotype modifications in the transformant strains were validated by PCR reactions using specific primers for the *CYP61* gene, zeocin or hygromycin B resistance cassettes (Table 1) and genomic DNA from the parental and transformant strains. The amplicons confirmed the *CYP61* gene interruption (Figure 5). However, as strain UCD 67–385 is diploid [30] and we were able to detect a *CYP61* wild-type allele, the resulting strain 385-*cyp61*^*hph*^ is heterozygous (385-*CYP61*/*cyp61*^*hph*^). For this reason, strain 385-*CYP61*/*cyp61*^*hph*^ was transformed with the linearized plasmid pBS-cyp61/Zeo obtaining the *cyp61*^*-*^ homozygote mutant strain 385-*cyp61*^*hph*^/*cyp61*^*zeo*^ (Figure 5). The ploidy levels of strains CBS 6938 and AVHN2 are unknown; based on random mutagenesis experiments and by transformation of carotenogenic genes performed at our laboratory [21,31], we estimate that these strains are aneuploid. In these cases, the PCR-based genotype analysis determined that a unique *CYP61* gene copy was mutated in strains CBS*-cyp61*^*hph*^ and Av2*-cyp61*^*zeo*^ (Figure 5), indicating that these strains are hemizygous, so a second transformation event was not necessary in these mutants. Interestingly, a clear difference in the color phenotype could be distinguished among all the *cyp61*^*-*^ mutants and their corresponding parental strains, indicating alterations in carotenoid biosynthesis (see below).

### *CYP61* gene mutant phenotype evaluation: ergosterol and carotenoid production

To analyze and compare the *cyp61*^*-*^ mutant phenotypes, the seven strains UCD 67–385, 385-*CYP61*/*cyp61*^*hph*^, 385-*cyp61*^*hph*^/*cyp61*^*zeo*^, CBS 6938, CBS*-cyp61*^*hph*^*,* AVHN2 and Av2*-cyp61*^*zeo*^ were cultivated in YM complete medium for 5 days at 22°C with constant agitation. Growth was measured by the culture absorbance at 600 nm, and samples were taken after 24, 72 and 120 h of cultivation. The samples were processed to determine the yeast dry weight and to extract sterols, carotenoids and RNA as described in the Materials and Methods section.

**Figure 6:**
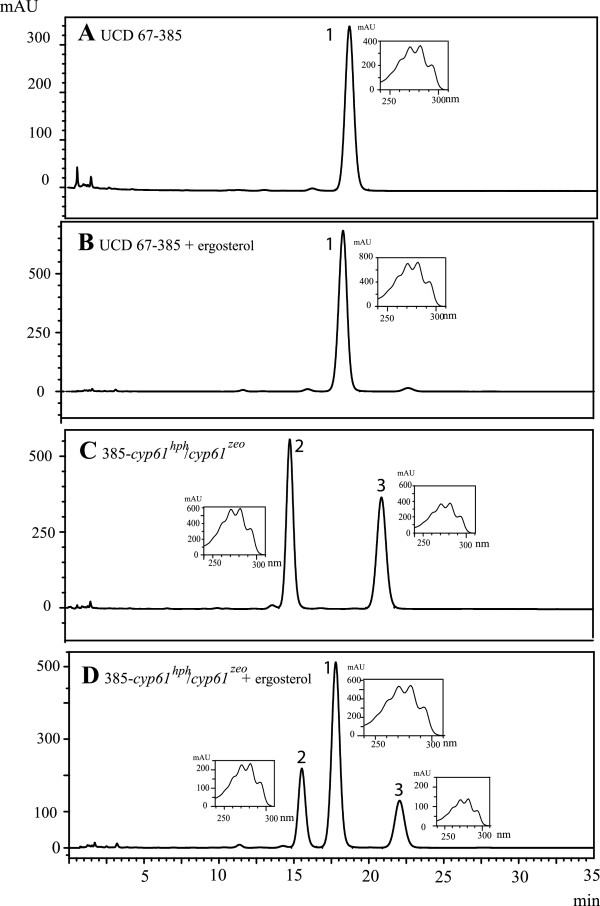
**RP**-**HPLC sterols analysis from UCD 67**–**385 and 385**-***cyp61***^***hph***^/***cyp61***^***zeo***^**strains.** Chromatograms (at 280 nm) correspond to sterols extracted from strains as described in the Materials and Methods section. Beside each peak (peaks Nº 1 to 3), the corresponding spectra were included. Sterols were analyzed from UCD 67–385 wild-type (**A**), UCD 67–385 wild-type co-injected with standard ergosterol (**B**), 385-*cyp61*^*hph*^/*cyp61*^*zeo*^ mutant (**C**) and 385-*cyp61*^*hph*^/*cyp61*^*zeo*^ co-injected with standard ergosterol. Ergosterol corresponds to peak Nº 1.

As in other species, the *CYP61* gene is involved in the ergosterol biosynthesis, so we evaluated the sterol production and composition in the *cyp61*^*-*^ mutants by RP-HPLC. Figure 6 shows representative chromatograms obtained from sterols extracted from strains UCD 67–385 and 385-*cyp61*^*hph*^/*cyp61*^*zeo*^, representing the parental and the *cyp61*^*-*^ mutant strains, respectively. In wild-type strains, we observed a predominant peak (peak 1) at the 280 nm channel at approximately 18 min with the ergosterol characteristic spectra (Figure 6A), and its identity was confirmed by co-injecting each sample with standard ergosterol (Figure 6B). On the other hand, in the analysis of the sterols from the homozygous and hemizygous *cyp61*^*-*^ mutants, two peaks were observed with retention times close to 15 (peak 2) and 21 min (peak 3) (Figure 6C, Table 3). These two peaks did also have the sterols characteristic spectra, but their retention time was different than that of ergosterol. This last observation was also confirmed by co-injecting the extracted sterols with standard ergosterol, resulting in three peaks at approximately 15, 18 and 22 min (Figure 6 D). Additionally, the identity of the sterols was determined by GC-MS (Additional file 1: Figure S1), confirming that wild-type strains produced mainly ergosterol and that the mutants instead accumulated ergosta-5,8,22-trien-3-ol and ergosta-5,8-dien-3-ol. Considering the relative abundance of each sterol obtained by GC-MS and RP-HPLC, peaks 2 and 3 in the RP-HPLC chromatogram from the *cyp61*^*-*^ mutant strain (Figure 6 C) should correspond to ergosta-5,8-dien-3-ol and ergosta-5,8,22-trien-3-ol, respectively.

**Table 3:** **Sterol composition according to their RP**-**HPLC profile of wild**-**type and *cyp61 X****.****dendrorhous *mutant strains** (**in mg**/**g dry yeast weight**)

	**Strains**								
	**UCD 67-385**			**385-*cyp61***^**(+/−)**^			**385**-***cyp61***^**(−/−)**^		
**Cultivation time** (**h**)	**24**	**72**	**120**	**24**	**72**	**120**	**24**	**72**	**120**
Ergosterol*	4.74±0.53	3.10±0.09	2.24±0.42	3.19±0.48	2.87±0.32	2.91±0.34	ND	ND	ND
Peak 2**	0.23±0.03	0.030±0.003	0.10±0.05	0.62±0.05	0.11±0.03	0.12±0.02	6.34±2.68	2.36±0.74	2.39±0.27
Peak 3***	0.19±0.04	ND	0.09±0.02	0.11±0.01	0.02±0.01	0.01±0.003	1.65±0.84	1.91±0.51	2.20±0.42
**Total Sterols**	**5**.**16**±**0**.**57**	**3**.**13**±**0**.**09**	**2**.**40**±**0**.**49**	**3**.**96**±**0**.**44**	**2**.**99**±**0**.**35**	**3**.**04**±**0**.**36**	**8**.**14**±**3**.**42**	**4**.**27**±**1**.**24**	**4**.**59**±**0**.**70**
	**Strains**								
	**CBS 6938**			**CBS***-****cyp61***^(−)^					
**Cultivation time** (**h**)	**24**	**72**	**120**	**24**	**72**	**120**			
Ergosterol*	3.31±0.60	2.39±0.56	2.37±0.11	ND	ND	ND			
Peak 2**	0.07±0.04	0.06±0.02	0.06±0.01	2.00±0.34	1.24±0.02	1.23±0.04			
Peak 3***	0.03±0.001	0.02±0.01	0.03±0.01	2.38±0.29	2.60±0.08	3.05±0.17			
**Total Sterols**	**3**.**45**±**0**.**56**	**2**.**41**±**0**.**59**	**2**.**46**±**0**.**11**	**4**.**38**±**0**.**61**	**3**.**85**±**0**.**1**	**4**.**28**±**0**.**21**			
	**Strains**								
	**AVHN2**			**AV2***-****cyp61***^(−)^					
**Cultivation time** (**h**)	**24**	**72**	**120**	**24**	**72**	**120**			
Ergosterol*	1.59±0.62	2.35±0.59	3.27±0.38	ND	ND	ND			
Peak 2**	ND	0.04±0.01	0.04±0.01	1.68±0.78	2.10±0.32	1.78±0.13			
Peak 3***	ND	ND	ND	1.39±0.82	2.27±0.18	2.39±0.52			
**Total Sterols**	**1**.**59**±**0**.**62**	**2**.**39**±**0**.**59**	**3**.**31**±**0**.**39**	**3**.**16**±**1**.**70**	**4**.**36**±**0**.**49**	**4**.**11**±**0**.**64**			

Table shows the mean values ± standard deviations of three independent experiments. Retention time: *: 18 min; **: 15 min; ***: 22 min. ND: Not detected.

Table 3 summarizes the sterol composition of the seven strains at different cultivation times. In general, when compared to the corresponding parental strain, the total sterol content was greater in the *cyp61*^*-*^ mutants. In addition, the sterols produced by the *cyp61*^*-*^ mutant strains corresponding to peaks 2 and 3 were at ratios of 55% and 44%, 32% and 68%, 48% and 52% in the 385-*cyp61*^*hph*^/*cyp61*^*zeo*^, CBS-*cyp61*^*hph*^, Av2-*cyp61*^*zeo*^ strains, respectively. In the heterozygous strain 385-*CYP61*/*cyp61*^*hph*^, the main sterol produced was ergosterol.

**Figure 7:**
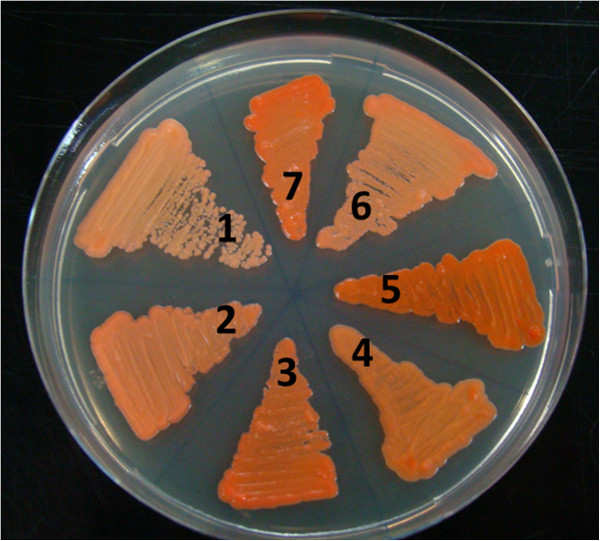
**Color phenotype of *cyp61 *mutant and wild**-**type strains.** Cultures in solid YM complete media of strains UCD 67–385 (**1**), 385-*CYP61*/*cyp61*^*hph*^ (**2**), 385-*cyp61*^*hph*^/*cyp61*^*zeo*^ (**3**), AVHN2 (**4**)*,* Av2*-cyp61*^*zeo*^ (**5**), CBS 6938 (**6**) and CBS*-cyp61*^*hph*^ (**7**).

**Figure 8:**
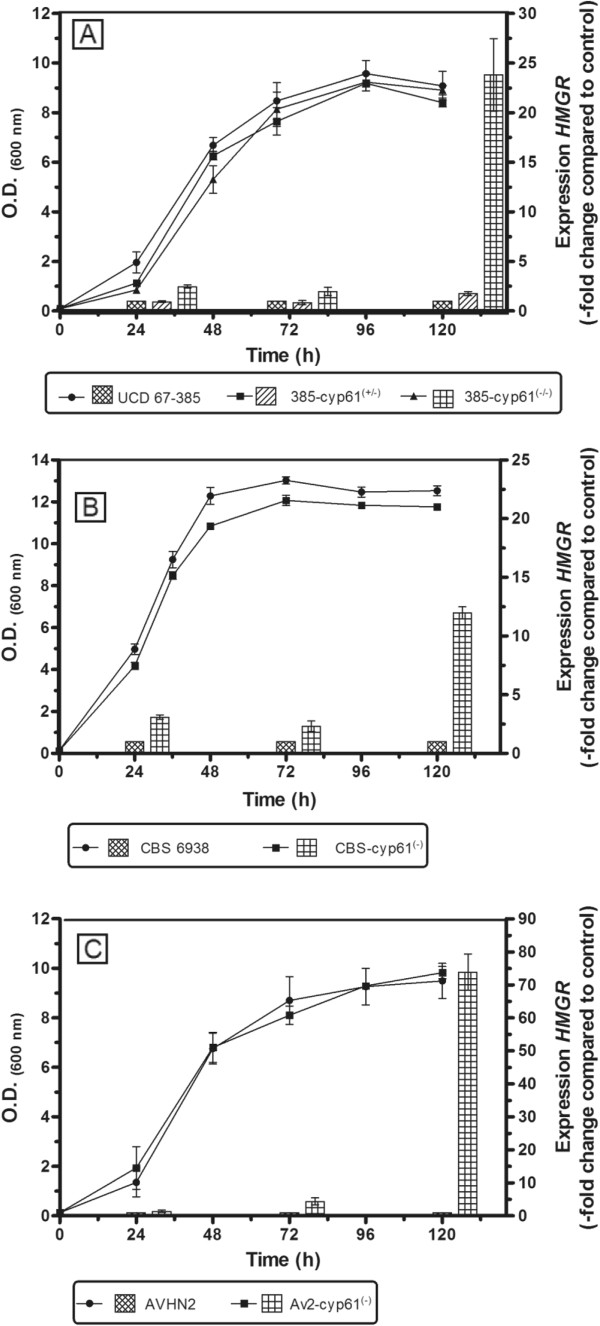
**RT**-**qPCR expression analysis of the *HMGR *gene along the growth curve in wild**-**type and *cyp61***^*-*^**mutant strains.** The HMGR gene expression in the mutant strains was determined with respect to the control (wild-type strain). **A**) Strains UCD 67–385, 385-*CYP61*/*cyp61*^*hph*^ and 385-*cyp61*^*hph*^/*cyp61*^*zeo*^**B**) CBS 6938 and CBS*-cyp61*^*hph*^. **C**) AVHN2 and Av2*-cyp61*^*zeo*^. Values are the mean ± standard error of three independent experiments.

A visible change in the color of the *cyp61*^*-*^ mutants was evident when compared to their corresponding parental strain (Figure 7). The first ones had a more intense red color, suggesting that the mutant strains produced more carotenoids. This observation was confirmed by carotenoid extraction and quantification from the seven strains after 24, 72 and 120 h of cultivation; the pigment composition was analyzed by RP-HPLC (Table 4). The *cyp61*^*-*^ mutants produced more carotenoids than their corresponding parental strains without other major alterations in their composition. In all cases, the maximum carotenoid content was reached after 120 h of cultivation, which coincides with the late stationary phase of growth (Figure 8). In general at this time, the major differences in total carotenoid content were observed among the analyzed strains. The total carotenoid contents relative to the parental strains after 24, 72 and 120 h of cultivation, respectively, were as follows: 126%, 132% and 101% in strain 385-*CYP61*/*cyp61*^*hph*^; 179%, 217% and 191% in strain 385-*cyp61*^*hph*^/*cyp61*^*zeo*^; 116%, 153% and 138% in strain CBS*-cyp61*^*hph*^ and 100%, 141% and 134 % in strain Av2*-cyp61*^*zeo*^ (Table 4).

**Table 4:** **Carotenoid composition of wild**-**type and *cyp61 X****.****dendrorhous *mutant strain** (**in ppm**)

	**Strains**								
	**UCD 67-385**			**385-*cyp61***^**(+/−)**^			**385-*cyp61***^**(−/−)**^		
**Cultivation time** (**h**)	**24**	**72**	**120**	**24**	**72**	**120**	**24**	**72**	**120**
**Astaxanthin**	52.6±22.3	26.3±2.7	224.0±42.1	89.1±13.4	34.9±5.1	223.7±8.6	126.5±31.0	49.8±18.2	434.7±56.2
**Phoenicoxanthin**	ND	ND	ND	ND	ND	ND	ND	ND	ND
**Cantaxanthin**	ND	ND	13.4±3.3	ND	ND	ND	ND	ND	ND
**HO**-**keto**-**γ**-**carotene**	ND	1.0±0.5	ND	ND	1.9±0.3	ND	ND	2.2±1.3	ND
**HO**-**keto**-**torulene**	2.6±1.1	1.1±0.2	30.1±6.7	ND	ND	35.5±1.0	ND	ND	62.1±7.3
**Keto**-**γ**-**carotene**	8.0±4.9	2.7±1.4	7.8±1.9	ND	1.2±0.6	9.7±1.0	ND	5.7±2.9	21.4±7.9
**HO**-**echinenone**	1.8±0.6	1.2±0.9	2.6±0.5	ND	2.6±0.5	9.2±0.4	ND	3.6±1.6	15.6±4.4
**Echinenone**	ND	ND	2.0±0.4	ND	ND	ND	ND	ND	ND
**Lycopene**	4.0±2.0	ND	ND	ND	1.4±0.7	1.1±1.0	ND	4.3±1.9	ND
**γ**-**carotene**	ND	0.2±0.03	2.7±0.5	ND	ND	ND	ND	0.8±0.4	ND
**β**-**carotene**	1.1±0.5	0.8±0.3	2.7±1.1	ND	1.7±1.0	6.3±0.8	ND	4.8±3.5	15.8±9.1
**Total carotenoids**	**70**.**7**±**26**.**9**	**36**.**1**±**8**.**6**	**290**.**1**±**53**.**4**	**89**.**1**±**13**.**4**	**47**.**6**±**7**.**1**	**293**.**7**±**9**.**1**	**126**.**5**±**31**.**0**	**78**.**2**±**26**.**2**	**555**.**1**±**75**.**2**
	**Strains**								
	**CBS 6938**			**CBS***-****cyp61***^(−)^					
**Cultivation time** (**h**)	**24**	**72**	**120**	**24**	**72**	**120**			
**Astaxanthin**	32.1±11.2	202.0±17.7	324.2±6.7	62.8±5.4	313.5±24.1	429.3±26.5			
**Phoenicoxanthin**	13.7±8.0	8.1±2.1	ND	ND	ND	ND			
**Cantaxanthin**	ND	ND	ND	ND	ND	ND			
**HO**-**keto**-**γ**-**carotene**	2.9±1.4	9.5±0.6	ND	2.7±2.0	ND	12.2±10.5			
**HO**-**keto**-**torulene**	ND	20.1±3.6	25.6±12.4	ND	76.4±8.3	72.8±18.0			
**Keto**-**γ**-**carotene**	9.8±4.6	32.8±4.6	29.8±0.45	7.1±0.8	50.2±3.5	33.0±2.97			
**HO**-**echinenone**	1.4±0.8	21.9±5.2	15.7±0.6	3.9±0.1	24.1±1.6	18.8±1.0			
**Echinenone**	ND	ND	ND	ND	ND	ND			
**Lycopene**	16.0±1.3	ND	ND	11.9±4.9	3.2±0.5	2.9±0.1			
**γ**-**carotene**	2.4±2.0	7.3±1.6	7.6±0.5	ND	8.8±0.2	15.3±1.7			
**β**-**carotene**	0.4±0.2	33.2±6.8	20.4±0.7	1.8±1.2	41.8±4.2	31.2±1.4			
**Total carotenoids**	**78**.**9**±**21**.**3**	**347**.**2**±**36**.**9**	**453**±**11**.**1**	**91**.**9**±**7**.**44**	**530**.**3**±**21**.**4**	**625**.**8**±**22**.**9**			
	**Strains**								
	**AVHN2**			**AV2***-****cyp61***^(−)^					
**Cultivation time** (**h**)	**24**	**72**	**120**	**24**	**72**	**120**			
**Astaxanthin**	15.2±0.8	116.5±7.0	131.8±20.6	16.3±6.1	118.0±59.2	143.0±64.8			
**Phoenicoxanthin**	ND	ND	ND	ND	ND	ND			
**Cantaxanthin**	ND	ND	ND	ND	ND	ND			
**HO**-**keto**-**γ**-**carotene**	ND	20.0±1.2	17.9±2.8	ND	25.3±7.8	36.8±16.7			
**HO**-**keto**-**torulene**	0.7±0.4	27.0±10.4	21.1±2.6	1.1±0.9	62.8±22.3	40.6±9.9			
**Keto**-**γ**-**carotene**	3.0±1.07	ND	ND	1.7±0.7	13.1±9.25	ND			
**HO**-**echinenone**	2.1±0.6	10.9±5.7	9.9±0.9	ND	9.3±7.3	13.6±2.6			
**Echinenone**	ND	ND	ND	ND	ND	ND			
**Lycopene**	1.4±1.0	ND	ND	ND	4.0±2.5	ND			
**γ**-**carotene**	ND	0.8±0.1	ND	ND	2.2±1.7	1.1±0.9			
**β**-**carotene**	1.0±0.5	19.7±12.0	12.0±2.9	1.9±0.9	25.4±7.6	20.4±4.7			
**Total carotenoids**	**24**.**9**±**2**.**8**	**195**.**3**±**33**.**7**	**193**.**4**±**19**.**0**	**25**.**0**±**6**.**9**	**274**.**6**±**24**.**1**	**258**.**6**±**76**.**7**			

Table shows the mean values ± standard deviations of three independent experiments.

ND: Not detected.

### Expression analysis of the *HMGR* gene

In *Schizosaccharomyces pombe, Cryptococcus neoformans* and mammalian cells, the expression of the HMG-CoA reductase encoding gene (*HMGR*) is regulated by sterols [11,32,33]. In *X. dendrorhous*, only one *HMGR* gene [GenBank: AJ884949] has been identified, and its deduced amino acid sequence shares 58% identity and 73.4% similarity with HMG1, one of the two HMG-CoA reductases in *S. cerevisiae*[34]. Thus, it is possible that the *X. dendrorhous cyp61*^*-*^ mutants have a higher *HMGR* transcript level, which could explain the increase in carotenoid content. We quantified the *HMGR* mRNA level by RT-qPCR at different timepoints on the growth curve of the seven analyzed strains. Figure 8 shows the relative expression of this gene normalized to the housekeeping beta-actin gene [35]. The *HMGR* gene expression pattern was different between the *cyp61*^*-*^ mutants and their parental strains. While its expression is relatively constant in the wild-type strains, its expression in the 385-*cyp61*^*hph*^/*cyp61*^*zeo*^, CBS*-cyp61*^*hph*^ and Av2*-cyp61*^*zeo*^ mutants increases along the growth curve, reaching expression levels almost 23, 12 and 73 times higher, respectively, than the wild-type strains after 120 h of cultivation. At 24 and 72 h of cultivation, the expression of this gene was between 2 and 5 times higher in the 385-*cyp61*^*hph*^/*cyp61*^*zeo*^, CBS*-cyp61*^*hph*^ and Av2*-cyp61*^*zeo*^ strains than in the respective parental strains (Figure 8).

## Discussion

Cytochrome P450 monooxygenases are involved in the oxidative metabolism of an enormous diversity of substrates, taking part in primary, secondary and xenobiotic metabolism. CYP51 and CYP61 are structurally and functionally conserved fungal P450s involved in membrane ergosterol biosynthesis [36], and the role of CYP61 as a C22-desaturase in fungal membrane sterol synthesis has been elucidated in *S. cerevisiae*[24] and *Candida glabrata*[37]. In this study, we isolated and characterized a gene, *CYP61*, from *X. dendrorhous* that has nine exons, encodes a putative 526-residue polypeptide and shares significant similitude and identity with the C22-sterol desaturase from *S. cerevisiae*[25]. We could predict several P450 characteristic secondary structural elements, and we identified three residues in CYP61 that are completely conserved in P450s. Together, these observations support the hypothesis that the *X. dendrohous CYP61* gene encodes the cytochrome P450 CYP61.

As in other organisms [25], the *CYP61* gene is not essential for the *X. dendrorhous* viability, even though we demonstrated that it is involved in ergosterol biosynthesis. Disruption of the *CYP61* gene prevents ergosterol biosynthesis and leads to the accumulation of other intermediary sterols including ergosta-5,8-dien-3-ol and ergosta-5,8,22-trien-3-ol. Contrary to our findings, the specific mutation of *ERG5* in *S. cerevisiae* results in the predominant accumulation of ergosta-5,7-dien-3-ol, although the C22-desaturase substrate is ergosta-5,7,24-trien-3-ol [25,38]. Like in *X. dendrohous*, ergosta-5,8,22-trien-3-ol accumulation has been observed in other fungi, such as *C. neoformans*, after the inhibition of the *ERG6*-encoding enzyme [39] and in nystatin-resistant *Neurospora crassa* strains that are unable to produce ergosterol [40]. Although our second found intermediary, ergosta-5,8-dien-3-ol, is an atypical sterol, it has been detected in fungi strains that are unable to synthetize ergosterol that in turn are resistant to fungicidal polyenes, such as nystatin and primaricin; polyenes bind ergosterol in the fungal cell membrane, creating channels that disrupt the transmembrane potential and its functions [41]. This phenomenon was observed in a nystatin-resistant *S. cerevisiae* strain [42] and primaricin-resistant *Aspergillus nidulans* strains [43]. Clearly, these observations and our results indicate the existence of alternative sterol biosynthesis pathways, which require further studies.

Because the *cyp61*^*-*^ mutant strains were viable without significant changes in their growth (at least in our experimental conditions), ergosta-5,8,22-trien-3-ol and/or ergosta-5,8-dien-3-ol may play roles similar to ergosterol in the *X. dendrorhous* cell membrane. Finally, even though the *cyp61*^*-*^ mutant strains were not able to produce ergosterol, their sterol content was higher compared to the corresponding parental strains, suggesting an ergosterol-mediated feedback regulatory mechanism in the sterol biosynthesis pathway of *X. dendrorhous*.

In addition to the alterations in sterol content and composition, the *cyp61*^*-*^ mutant *X. dendrorhous* strains exhibited color phenotypes dissimilar to their parental strains (Figure 7). Carotenoid analyses revealed that the mutant strains produced more carotenoids (Table 4), demonstrating that the *CYP61* gene mutation affected carotenoid biosynthesis. Major differences were observed after 72 and 120 h of culture, which coincide with the early and late stationary phases of growth (Figure 8). Wozniak and co-workers reported that maximum expression levels of carotenogenic genes are reached by the end of the exponential and beginning of the stationary phase of *X. dendrorhous* growth [44], coinciding with the induction of carotenogenesis [45]. It is expected that greater differences in the carotenoid content would be observed once carotenogenesis is induced.

Similar to our results, other studies have demonstrated an increase in astaxanthin production in *Phaffia rhodozyma* (anamorphic state of *X. dendrorhous*) when the ergosterol levels were reduced by fluconazole treatment [46]. A possible explanation for the increased carotenoids in the *cyp61*^*-*^ mutants could be the greater availability of carotenoid precursors in absence of the ergosterol negative feedback regulation. This reasoning is also supported by the fact that in the *cyp61*^*-*^ mutants, the total sterol content was also increased. For example, supplementation of *P. rhodozyma* cultures with MVA resulted in an increase in carotenoid production [47]. Likewise, deletion of the squalene synthase-encoding gene (*ERG9*) in combination with the overexpression of the catalytic domain of HMGR in a recombinant *C. utilis* strain that produces carotenoids caused an increase of in lycopene biosynthesis [48].

IPP is the isoprenoid building block; in most eukaryotes, it is derived from the MVA pathway [10]. Many of the regulatory aspects of isoprenoid biosynthesis involve elements of this pathway; the expression of *HMGR* (Figure 1) is a critical regulatory step [49]. The alteration of *HMGR* expression in the *X. dendrorhous cyp61*^*-*^ mutants could explain the increased carotenoid and sterol content. We quantified the *HMGR* transcript levels, and at all of the growth phases analyzed, it was greater than in the corresponding parental strain. Similarly, an increase in the *HMGR* transcript level corresponded to an increase in the carotenoid content when the fungus *Blaskelea tripora* was treated with ketoconazole, which is a specific ergosterol biosynthesis inhibitor [50]. In *S. cerevisiae*, HMG-CoA reductase is encoded by two isogenes, *HMG*1 and *HMG*2, and the expression of *HMG*1 is controlled at the transcriptional level by ergosterol [26]. The overexpression of *HMG1* combined with ketoconazole treatment in a *S. cerevisiae* recombinant strain resulted in an increase in beta-carotene production [51]. Finally, our results are similar to those reported in the astaxanthin over-producing *X. dendrorhous* mutant strain with lower ergosterol and a higher *HMGR* transcript level than the parental strain after 72 h of cultivation [46]. However, the astaxanthin over-producing strain was obtained by random chemical mutagenesis, while we specifically blocked ergosterol biosynthesis by disrupting the *CYP61* gene.

## Conclusions

In conclusion, the *CYP61* gene disruption in *X. dendrorhous* prevents the synthesis of ergosterol without affecting the growth of the yeast under the experimental conditions used in this work. The *cyp61*^*-*^ mutant strains accumulate ergosta-5,8,22-trien-3-ol and ergosta-5,8-dien-3-ol that may fulfill some of the ergosterol roles in the cell. In addition, our results strongly suggest that by a feedback regulatory mechanism, ergosterol regulates the synthesis of sterols and carotenoids in the astaxanthin-producing yeast *X. dendrorhous,* being the *HMGR* gene expression, one of its targets.

## Methods

### Microorganisms, plasmids, media, and enzymes

The strains and plasmids that were used or created in this work are listed in Table 2. The wild-type UCD 67–385 *X. dendrorhous* strain was used for cDNA library construction and genomic *CYP61* gene amplification. *E. coli* DH-5α was used as a host for plasmid propagation.

*X. dendrorhous* strains were grown at 22°C with constant agitation in YM medium (1% glucose, 0.3% yeast extract, 0.3% malt extract and 0.5% peptone). Yeast transformant selection was performed on 1.5% agar YM-plates supplemented with 10 μg/ml hygromycin B and/or 15 μg/ml zeocin. *E. coli* strains were grown with constant agitation at 37°C in Luria-Bertani (LB) medium supplemented with 100 μg/ml ampicillin for plasmid selection and 40 μl of a 2% solution of X-gal (5-bromo-4chloro-3-indolyl-β-D-galactopyranoside) for recombinant clone selection [52]. Recombinant clones bearing the plasmids with the hygromycin B or zeocin resistance cassettes were selected by direct colony PCR with primers specific for each cassette [21,31]. The zeocin resistance cassette was constructed in the same way as the hygromycin B resistance cassette [31] using the *Sh ble* gene from *Streptoalloteichus hindustanus*[53,54]. The *Taq* DNA polymerase (pol), restriction enzymes, Klenow polymerase and M-MLV reverse transcriptase were purchased from Promega, and the *Pfu* DNA pol was purchased from Invitrogen.

### DNA amplification and sequence analysis

The oligonucleotides designed for this study (Table 1) were purchased from Alpha DNA or from Integrated DNA Technologies. PCR reactions were performed in a DNA thermal cycler 2400 (Perkin-Elmer) in a final volume of 25 μl containing 2 U of *Taq* DNA pol, 2.5 μl of 10X *Taq* buffer, 0.5 μl of 10 mM dNTPs, 1 μl of 50 mM MgCl_2_, 1 μl of each primer (25 μM) and 10 to 20 ng of template DNA. In general, the amplification protocol was as follows: initial denaturation at 95°C for 3 min; 35 cycles of denaturation at 94°C for 30 s, annealing at 55°C for 30 s, and synthesis at 72°C for 3 min; and a final extension step at 72°C for 10 min. Samples were kept at 4°C until checked by 0.8% agarose gel electrophoresis in TAE buffer containing 0.5 μg/ml ethidium bromide [52]. DNA for sequencing or plasmid construction was purified from gels with glass milk [55].

Nucleotide sequences were obtained from an ABI 3100 Avant genetic analyzer using the BigDye terminator v3.1 kit (Applied Biosystems). DNA sequences were analyzed with Vector NTI Suite 10 (Informax), CLUSTAL W 1.8 and programs available at the NCBI web site. Protein sequence analyses were performed with programs available at http://www.ch.embnet.org/software/TMPRED_form.html[56], http://www.ebi.ac.uk/InterProScan/[57] and http://www.cyped.uni-stuttgart.de/[58].

### Cloning of the X*. dendrorhous CYP61* gene and plasmid construction

Our group has partially sequenced the genome of the wild-type UCD 67–385 *X. dendrorhous* strain by two Next Generation Sequencing (NGS) systems. Our collection of scaffolds covers approximately 95% of the haploid genome of the yeast. We used the CLC Genomics Workbench 5 for genome analyses. BLAST analyses allowed us to identify the *X. dendrorhous CYP61* gene, and primers were designed from its sequence (Table 1).

The pBS-gCyp61 plasmid (Figure 4) was generated by inserting a 4,224 bp PCR-amplified DNA fragment encoding the *CYP61* gene into the *Eco*RV site of pBluescript SK- plasmid. The DNA fragment was amplified using the primer set CYP61up2.F + CYP61dw2.R (Table 1) and genomic DNA of the UCD 67–385 wild-type strain as template. Plasmids pBS-cyp61/Hyg and pBS-cyp61/Zeo were created by cloning the hygromycin B and the zeocin resistance cassettes, respectively, into the *Eco*RV site of plasmid pBS-cyp61 (Figure 4). Plasmid pBS-cCyp61, bearing the cDNA of the *CYP61* gene, was obtained from a *X. dendrorhous* cDNA library made with the pBluescript II XR cDNA library construction kit (Stratagene) [31].

### *X. dendrorhous* transformation

*X. dendrorhous* transformation was performed by electroporation according to [59] and [60]. Electrocompetent cells were prepared from an exponential culture (OD_600nm_ = 1.2), grown in YM medium and electroporated using a BioRad gene pulser × cell with PC and CE modules under the following conditions: 125 mF, 600 Ω, 0.45 kV. Transformations were performed using 1 to 5 μg of linear donor DNA prepared by cutting pBS-cyp61/Hyg or pBS-cyp61/Zeo with *Xba*I. The transformant strains were identified as *X. dendrorhous* by analysis of the ITS1, 5.8 rRNA gene and ITS2 DNA sequences [61]. The transformant strains were identified as *X. dendrorhous* by analysis of the ITS1, 5.8 rRNA gene and ITS2 DNA sequences [61].

### Phenotypic analyses of *cyp61* mutant strains

To compare the phenotypic differences between wild-type and *CYP61* mutant strains, phenotypic analyses were performed. The strains were grown in YM medium, and growth curves were constructed including the analyses of total carotenoid yield and composition, ergosterol production and relative mRNA expression of the *HMGR* gene at three timepoints. For these analyses, the seven *X. dendrorhous* strains (UCD 67–385, 385-cyp61^(+/−)^, 385-cyp61^(−/−)^, CBS 6938, CBS-cyp61^(−)^, AVHN2 and Av2-cyp61^(−)^) were cultivated in triplicate 600 ml YM cultures in Erlenmeyer flasks at 22°C with constant agitation. The yeast growth was determined by the OD at 600 nm, which was measured in V-630 UV–vis Spectrophotometer from JASCO. Culture samples of 75 ml were taken after 24, 72 and 120 h of growth and segregated for analysis as follows: 5 ml to determine the dry weight of the yeast, 30 ml for RNA, 30 ml for pigment and 10 ml for sterol extractions. In each case, the cell pellet was washed with distilled water, frozen with liquid nitrogen and stored at −80°C until further processing.

### Carotenoid extraction and RP-HPLC

Carotenoids were extracted from cellular pellets according to the acetone extraction method [62]. Total carotenoids were quantified by absorbance at 465 nm using an absorption coefficient of A_1%_ = 2,100 and normalized to the dry weight of the yeast. Carotenoids were separated by RP-HPLC using a reverse phase RP-18 Lichrocart125-4 (Merck) column with acetonitrile: methanol: isopropyl (85:10:5 v/v) as the mobile phase with a 1 ml/min flux under isocratic conditions. The elution spectra were recovered using a diode array detector, and carotenoids were identified by their spectra and retention time according to standards.

### Sterol extraction and identification

Sterol extraction was adapted from [63] and [64]. Briefly, 4 g of KOH and 16 ml of 60% (v/v) ethanol/water were added to the cell pellets, which were mixed and saponified at 80 ± 2°C for 2 h. Non-saponificable sterols were extracted with 10 ml of petroleum and dried. Sterols were separated by RP-HPLC with a C-18 column, using methanol/water (97:3, v/v) as the mobile phase at 1 ml/min. The elution spectra were recovered using a diode array detector, and sterols were visualized in the 280 nm channel. Standard ergosterol was purchased at Sigma-Aldrich (catalogue number 57-87-4). Sterols were quantified spectrophotometrically at 280 nm [65]. The identification of the sterols was performed by an external service (Corthorn Quality; http://www.corthorn.cl/) by GC/MS (Agilent 5970N gas chromatographer/Agilent 5890N mass spectrometer). An RTX5 sil MS (Restk) 30 m × 250 μm × 0.25 μm column was used with the following oven conditions: 270°C for 10 s, raised to 280°C at 30°C/min and maintained for 2 min. The injector temperature was 270°C, and the ion source was kept at 70 eV. Helium was used as the mobile phase with a flux rate of 1 ml/min. For sterol identification the NIST Standard Reference Database 1A (NIST/EPA/NIH Mass Spectral Library (NIST 08) and NIST Mass Spectral Search Program version 2.0f, was used (http://www.nist.gov/srd/).

### RNA extraction, single strand DNA synthesis and RT-qPCR

Total RNA extraction from the cell pellets was performed via mechanical rupture with 0.5 mm glass beads (BioSpec) and shaking in a vortex apparatus for 10 min followed by the addition of Tri-Reagent (Ambion). The lysate was incubated for 10 min at room temperature, and 150 μl of chloroform per ml of Tri-Reagent was added. The aqueous phase was recovered after centrifugation for 5 min at 4,000 x g. Two consecutive extractions with acidic phenol:chloroform (1:1) were performed, and the RNA was precipitated by adding two volumes of isopropanol and incubating at room temperature for 10 min. The RNA was washed with 75% ethanol, suspended in RNase-free H_2_O and quantified by absorbance determination at 260 nm in V-630 UV–vis Spectrophotometer from JASCO.

The synthesis of cDNA was performed according to the M-MLV reverse transcriptase (Invitrogen) manufacturer’s protocol, with 5 μg of total RNA in a final volume of 20 μl. The determination of the relative gene expression levels was performed in an Mx3000P quantitative PCR system (Stratagene) using 1 μl of the reverse transcription reaction, 0.25 μM of each primer (Table 1) and 10 μl of the SensiMix SYBR Green I (Quantace) kit in a final volume of 20 μl. The Ct values obtained were normalized to the respective value of the beta-actin, *ACT* [Genbank: X89898.1] [66] and later expressed as a function of the control conditions using the ΔΔCt algorithm [35].

## Competing interests

The authors declare no competing financial or any non-financial interests.

## Authors’ contributions

JA conceived the study, participated in its design and coordination. JA carried out the *cyp61* gene isolation, sequence analysis and *X. dendrorhous* transformation. IL performed the gene expression, pigment and ergosterol extraction analyses. MSG did the genomic transformants analyses and SB accomplished the growth curves of wild-type and *cyp61* mutant strains. DS participated in DNA sequencing. PM-M participated in the gene expression analyses. MB contributed in the study design. VC participated in the experiment design and coordination. JA, MB, VC drafted the manuscript. All authors read and approved the final manuscript.

## Supplementary Material

Additional file 1 **Figure S1.** GC-MS analysis of sterols from wild-type and cyp61 X. *dendrorhous* mutant strain. GC profiles of sterols (peaks Nº 1, 2 and 3) from UCD 67–385 (panel A) and 385-*cyp61*^*(−/−)*^ (panel B) strains. Sterols structures were identified according to their retention times and mass spectra (NIST Standard Reference Database). Panels C, D and E show the sample (in red) and Database (in blue) mass spectra: ergosterol (peak Nº 1, panel C), ergosta-5,8,22-trien-3-ol (peak Nº 2, panel D) and ergosta-5,8-dien-3-ol (peak Nº 3, panel E). Click here for file
