# Supplementary figures and images for: Enhancement of carotenoid production by disrupting the C22-sterol desaturase gene (CYP61) in Xanthophyllomyces dendrorhous
Source: BMC Microbiol. 2012 Oct 18;12:235. doi: 10.1186/1471-2180-12-235 (PMC3552872; doi:10.1186/1471-2180-12-235)

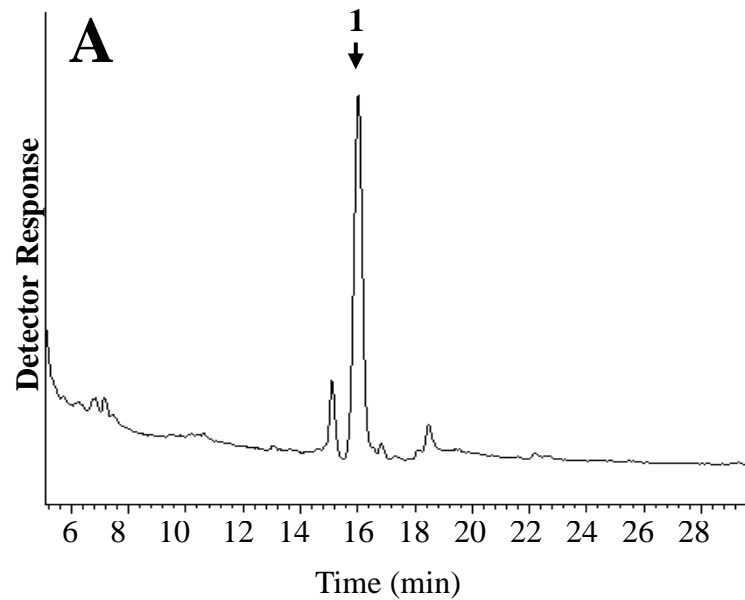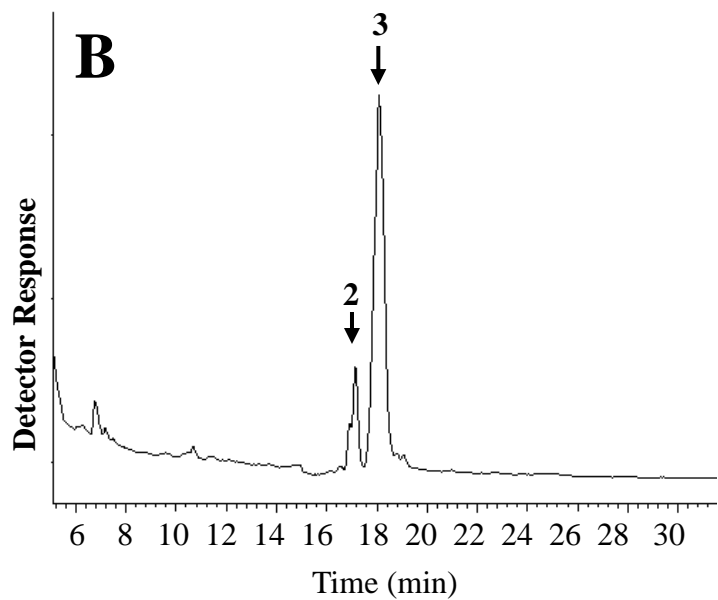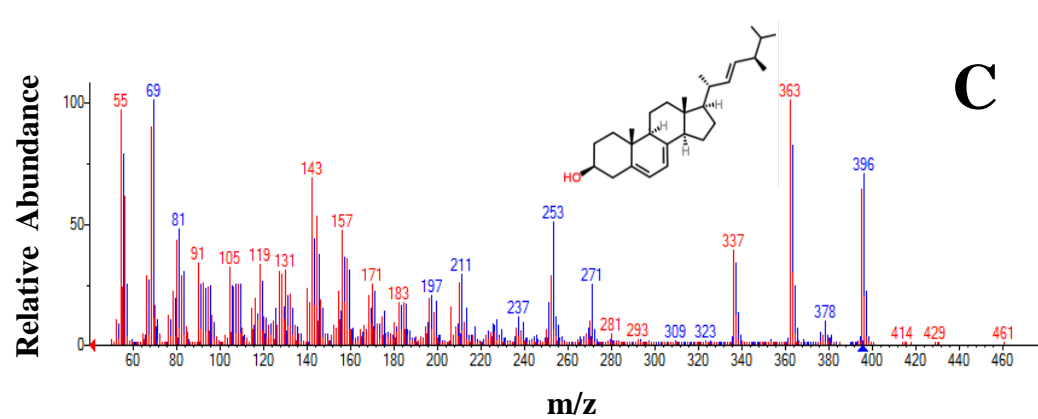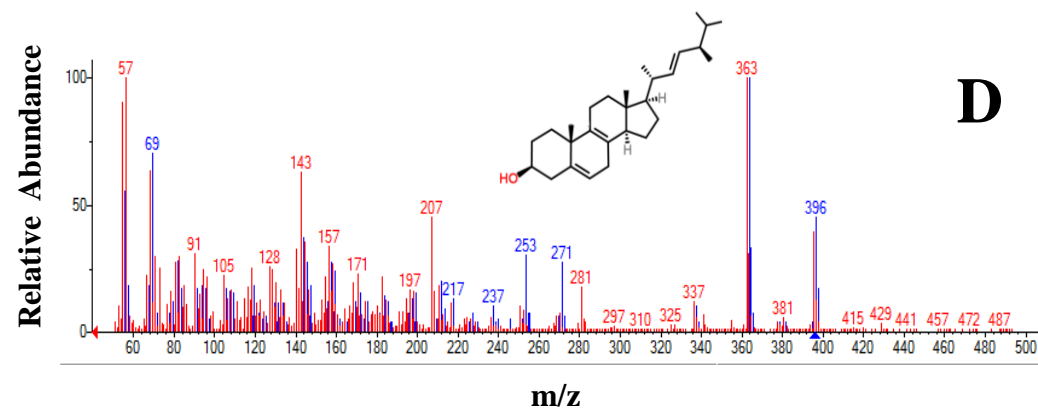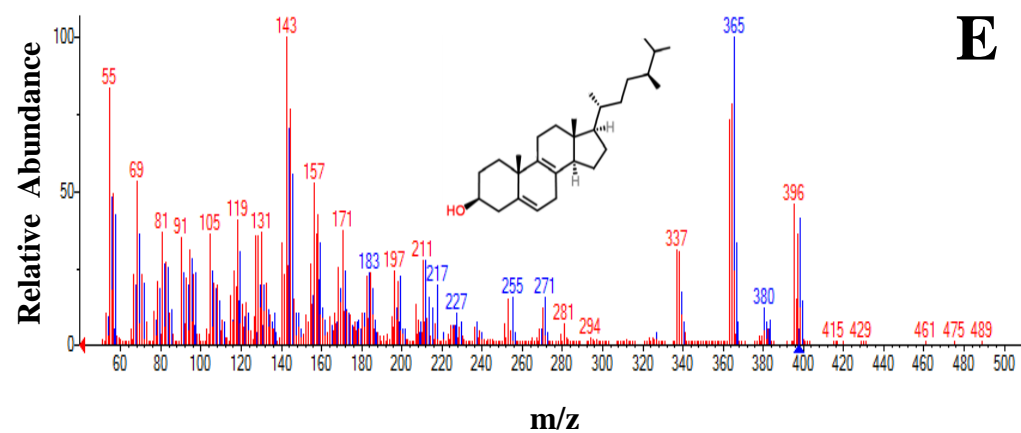

Supplement: Additional file 1 — Figure S1. GC-MS analysis of sterols from wild-type and cyp61 X. dendrorhous mutant strain. GC profiles of sterols (peaks Nº 1, 2 and 3) from UCD 67–385 (panel A) and 385-cyp61(−/−) (panel B) strains. Sterols structures were identified according to their retention times and mass spectra (NIST Standard Reference Database). Panels C, D and E show the sample (in red) and Database (in blue) mass spectra: ergosterol (peak Nº 1, panel C), ergosta-5,8,22-trien-3-ol (peak Nº 2, panel D) and ergosta-5,8-dien-3-ol (peak Nº 3, panel E). [file 1471-2180-12-235-S1.pdf]
